# PREDICTING TRANSDIAGNOSTIC PSYCHOPATHOLOGY FROM INDICES OF AGING IN THE HUMAN STRUCTURAL CONNECTOME

**DOI:** 10.1093/geroni/igz038.1261

**Published:** 2019-11-08

**Authors:** James Madole, James W Madole, Simon R Cox, Colin R Buchanan, Stuart J Ritchie, Mark E Bastin, Ian J Deary, Elliot M Tucker-Drob

**Affiliations:** 1 The University of Texas at Austin, Austin, Texas, United States; 2 The University of Edinburgh, Edinburgh, Scotland, United Kingdom; 3 King’s College London, London, England, United Kingdom

## Abstract

Imaging-derived indices of brain structure and white-matter connectivity evince steep declines with adult age and are robustly linked to neurological disease and a wide range of psychopathologies. Risk for psychopathology may be related to rapid structural brain aging, but the specific patterns of relations are not well documented. Using structural and diffusion MRI data from UK Biobank, we estimated a structural connectome for each participant (N = 3155), and used empirically-driven machine-learning algorithms to identify features of the connectome most susceptible to brain aging. In an age-homogenous hold-out sample of older adults, we score participants’ “connectome age” using the coefficients saved from the training sample. We examine associations between connectome age and both psychiatric symptom counts and polygenic risk scores for a range of psychiatric disease traits. This will be amongst the first and most comprehensive investigation of the extent to which psychopathology relates to signatures of structural connectome aging.

